# Transmetabolism: the non‐conformist approach to biotechnology

**DOI:** 10.1111/1751-7915.13691

**Published:** 2020-10-29

**Authors:** Juli Peretó

**Affiliations:** ^1^ Department of Biochemistry and Molecular Biology University of Valencia Institute for Integrative Systems Biology I2SysBio (University of Valencia‐CSIC) Darwin Bioprospecting Excellence SL, Parc Científic de la Universitat de València Paterna Spain

Dedicated to the memory of Arren Bar‐Even (1980‐2020), one of the most brilliant biochemists of our times.

Living beings are the result of a cocktail made with unknown doses of chance and necessity. Consider a thought experiment, in which we could rewind the ‘tape of life’ starting from the same initial conditions, what biochemical traits and cellular features would finally be the same as those we observe today? It is clear that what is real in biology is a subset of what is possible, and this issue has been discussed at different scales. Thus, structural and dynamic developmental constraints limit the space of solutions for animal bodies (Alberch, 1989), whereas physicochemical restrictions and historical contingencies shape the possible at the molecular level (Jacob, 1981). Meteorite analysis and many organic syntheses performed under prebiotic conditions indicate that the primitive Earth was home of a moderately complicated chemodiversity, including the most common biological building blocks – sugars, fatty acids, amino acids, nucleobases, etc. (Lazcano, 2018). In this period of chemical evolution, physicochemical constraints (i.e. thermodynamics and kinetics in a given environment) determined the origin and maintenance of the abiotic chemical landscape. The chemically possible was the scenario for the organization of the most simple and primitive biochemical systems: autocatalytic cycles for self‐maintenance of a set of building blocks, self‐reproduction of lipid vesicles, and self‐replication of genetic templates (Peretó, 2012). Presumably, all these cycles kicked off in the absence of catalysts or with the involvement of very simple and unspecific facilitators (e.g. mineral surfaces). The emergence of optimizable catalysts through natural selection (e.g. ribozymes) was a phase transition to a period of a more efficient and creative functional screening of the possible (de Duve, 2005). Diverse lines of evidence indicate that metabolic surveys of alternative sources of matter and energy were rapid and explosive, performed by the first microbial communities. Biogeochemical closing of the recycling of bioelements was a vital step for a sustainable and long‐term continuity of terrestrial life (Falkowski *et al*., 2008). Thus, the boundaries of the metabolically possible were expanding in parallel to the coevolution of life and the planet. For instance, after the emergence in some cyanobacterial ancestors of the enzymatic machinery able to extract electrons from water to feed the photoelectronic chain, molecular oxygen accumulated in oceans and afterwards in the atmosphere. Those microorganisms able to cope with this new‐to‐life compound took advantage of its reactivity and dramatically expanded the world of the metabolically possible: many new metabolites, including steroids, and processes (e.g. oxygen respiration) became available to life. Thus, recurrent patterns in actual cell metabolisms are the result of a long evolutionary exploration within the chemically constrained space of the possible solutions under specific yet changing conditions (de Lorenzo *et al*., 2014).

The beginnings of the scientific study of metabolism focused on model systems (e.g. yeast and skeletal muscle) and well‐defined physiological processes (e.g. alcohol fermentation and cellular respiration). Describing chemical transformations in cell‐free systems and the use of inhibitors, mutants or isotope labelling helped to draw the first metabolic pathways (Morange, 2020). More recently, functional inference from environmental metagenomes combined with experimental validation (e.g. metabolomics and fluxomics) has expanded the spectrum of known metabolisms, albeit the scrutiny of old model systems still delivers surprises, as for example sulphoglycolysis in *Escherichia coli* K12 (Denger *et al*., 2014). Public databases now offer beautiful meta‐metabolic maps, that is the cartography of the discovered metabolism. But an unknown fraction of unknown metabolisms remains to be unveiled. Increased efforts in bioprospecting and studying more biochemistry from environmental samples will continue to push the boundaries of the known (Tanner *et al*., 2017).

Evolution does not deliver fully optimal metabolic solutions, yet just those sufficient for survival and reproduction. The fuzzy discreteness of metabolic circuits has allowed the classical strategy of metabolic engineering: the pursuit of the enhancement of cell performances upgrading metabolite fluxes of native pathways, even by means of transplantation of enzymes or pathways wired within the metabolic core of a heterologous host. Thus, metabolic engineering navigates inside the possible, playing within the limits of biological and physicochemical trade‐offs: usually, it is not possible to improve one trait without worsening other cellular performances (Sheftel *et al*., 2013). In addition to these constraints, some sub‐optimal solutions in extant metabolisms are also the result of historical contingencies brought to the present biosphere by irreversible evolutionary trajectories. The almost universal stoichiometric architecture of the central metabolic pathways includes some decarboxylations that affect the global yield of carbon conservation. Take the case of the conversion of hexoses in acetyl CoA: first, the hexose yields pyruvate and then this metabolite undergoes oxidative decarboxylation to acetyl CoA. This metabolic strategy results in an unavoidable loss of 33% of the initial carbon as CO_2_. Experiments in prebiotic systems chemistry, exploring the origin of glycolytic intermediates, indicate an early emergence of this historically frozen transformation of C3 in C2 moieties (Coggins and Powner, 2017). Although evolutionary tinkering may improve the performance of the metabolic systems alleviating the carbon leakage (Fontaine *et al*., 1942; Schwender *et al*., 2004), in the context of microbial biotechnology, a relevant question is as follows: can hexoses be stoichiometrically metabolized to acetyl CoA without carbon loss?

The community of microbial biotechnologists is ready to foster a non‐conformist approach to metabolic engineering, I mean, a biotechnology that goes beyond nature’s subset of the chemically possible. Today, there are many successful attempts of rational design of unnatural pathways with new stoichiometric balances, as well as repurposed enzymes performing new‐to‐nature reactions, including the incorporation of non‐biogenic chemical elements to metabolism (Prather, 2019). Powerful computational algorithms that search for new theoretical stoichiometries, and experimental evolution, allowing an efficient empirical evaluation of the possible with the help of natural selection under artificial conditions, are key approaches for the non‐rational exploration of the chemically available to the biochemical factory. We can invent enzymatic arrangements that, as far as it is known by the scientific community, have not been reached by any natural system due to historical constraints in the evolutionary trajectories. Although sometimes imagination precedes discovery. This is the case of the reductive glycine pathway, proposed by the untimely disappeared biochemist Arren Bar‐Even as a hypothetical formatotrophic or autotrophic pathway (Bar‐Even *et al*., 2013), that in the real world drives full autotrophic growth in *Desulfovibrio desulfuricans* (Sánchez‐Andrea *et al*., 2020).

Motivated by the Nobel prize winner Frances H. Arnold, I would say that metabolism is amazing not only because what it does now, but for what it *can* do forced by our creativity and inspiration. For instance, non‐oxidative glycolysis (NOG) is an artificial pathway composed of natural enzymes from different phylogenetic origins and showing a complete carbon conservation without decarboxylation steps during the transformation of glucose in acetate (i.e. one mole of hexose delivers three moles of acetate without carbon loss). NOG functions in vitro and in vivo in *E. coli*, by the action of canonical glycolytic and the pentose phosphate pathway enzymes from the host in combination of an enzyme of the bifid shunt, namely a phosphoketolase from *Bifidobacterium adolescentis,* allowing a new rearrangement and recycling of carbon skeletons (Bogorad *et al*., 2013). There is a diversity of examples of unnatural pathways already described in the bibliography, but without leaving the topic of increasing carbon conservation in metabolism, the reader is addressed to a recent review by François *et al*. (2020). The long‐term strategy of all these efforts is the design of new efficient metabolic stoichiometries allowing the transformation of non‐edible feedstocks in biofuels or commodity chemicals with a reduction of CO_2_ emissions by cellular factories. There is still a long way from all those laboratory successes to their efficient industrial implementations.

The idea of designing new metabolic processes and catalysts adopting a non‐conformist attitude – going beyond the naturally evolved pathways and enzymes – is not completely new. For instance, Fessner and Walter (1992) proposed the term ‘artificial metabolism’ to refer to new *in vitro* combinations of purified natural enzymes performing unnatural chemical processes. What is now certainly innovative are the approaches, the tools and the engineering ideology under current synthetic biology projects. Thus, Fessner (2015) has introduced the term ‘systems biocatalysis’ with the meaning of ‘cell‐free artificial metabolism’. On the other hand, James U. Bowie and collaborators use ‘synthetic biochemistry’ referring to the reconstruction of metabolic pathways in vitro and the design of devices to re‐engineer the complex networks of cofactor recycling, enabling maximal theoretical yields of transformations (Korman *et al*., 2014; Opgenorth *et al*., 2014). Tobias J. Erb and coworkers have introduced the term ‘synthetic metabolism’ referring rationally designed, new‐to‐nature, pathways and enzymes beyond the natural repertoire (Erb *et al*., 2017). Also, the term ‘xenobiology’ has been used to refer to efforts, for instance, in the expansion of the genetic code with artificial nucleobases or reassigning codons to artificial amino acids, specifically in the context of the design of orthogonal biosystems (Schmidt, 2010). The assembly of novel pathways engineered to handle non‐biological elements (or ‘xenoelements’) has been christened as ‘neometabolism’ (Nieto‐Domínguez and Nikel, 2020), whereas the neologism ‘transmetabolism’ refers to the extension of biological networks beyond their ‘natural’ components as suggested by Nikel and de Lorenzo (2018). I prefer the term ‘transmetabolism’ for its obvious resonances with the current meaning of the word ‘transhumanism’. According to the Oxford English Dictionary, transhumanism is ‘the belief or theory that the human race can evolve beyond its current physical and mental limitations, especially by means of science and technology’. In the next decades, transmetabolism will allow us to reach innovative and useful chemical solutions never explored by evolution, and beyond our ‘current physical and mental limitations’. In fact, part of these limits has started to melt by searching reaction networks with algorithms, empowered by artificial intelligence, for the discovery of new unnatural pathways leading to (natural or unnatural) compounds of industrial interest (Lin *et al*., 2019). Furthermore, computer‐generated networks of potential chemistries may open a window to the past, enabling empirical tests of plausible prebiotic scenarios (Wołos *et al*., 2020). The smart fusion of dry and wet biochemistries is our best baggage for this fascinating journey through transmetabolism. I am convinced that – paraphrasing an apocryphal Carl Sagan quotation – somewhere in the space of the possible, an incredible biochemical transformation is waiting to be known.
